# Beneficial impact of physical activity on multiple sclerosis disability progression

**DOI:** 10.1136/jnnp-2025-336738

**Published:** 2025-09-23

**Authors:** Anna Karin Hedström, Tomas Olsson, Fredrik Piehl, Lars Alfredsson

**Affiliations:** 1 Department of Clinical Neuroscience, Karolinska Institutet, Stockholm, Sweden; 2 Institute of Environmental Medicine, Karolinska Institutet, Stockholm, Sweden; 3 Centre for Occupational and Environmental Medicine, Region Stockholm, Stockholm, Sweden

**Keywords:** MULTIPLE SCLEROSIS

## Abstract

**Background:**

Physical activity has been associated with neuroprotective and immunomodulatory benefits, potentially influencing long-term disability outcomes in multiple sclerosis (MS). However, longitudinal evidence on its role in modifying disease progression remains limited.

**Methods:**

We analysed 3284 individuals with incident relapsing-remitting MS from the Swedish Epidemiologic Investigation of MS. Participants were followed for up to 15 years through the Swedish MS Registry. Physical activity at diagnosis was categorised into low, moderate, moderate-high and high activity. The primary outcomes were time to confirmed disability worsening (CDW) and progression to Expanded Disability Status Scale (EDSS) 3 and 4. Changes in physical activity post diagnosis were analysed in a subsample (n=1724). Cox regression models were used to estimate HRs adjusting for clinical and lifestyle factors.

**Results:**

Higher levels of physical activity at diagnosis were associated with reduced risk of disability progression. Compared with low physical activity, the risk of CDW was reduced for moderate (HR 0.77, 95% CI 0.61 to 0.97), moderate-high (HR 0.71, 95% CI 0.54 to 0.94) and high physical activity (HR 0.64, 95% CI 0.46 to 0.90). Similar trends were observed for reaching EDSS 3 and EDSS 4. Increasing activity post-diagnosis was associated with more favourable outcomes, while physical activity before diagnosis showed no significant association with long-term disability progression.

**Conclusions:**

Our findings suggest that physical activity may beneficially influence disease progression in MS. The observed associations highlight the importance of maintaining regular physical activity for ongoing clinical benefits. Our results support including physical activity promotion as a component of standard MS care to optimise long-term outcomes.

**WHAT IS ALREADY KNOWN ON THIS TOPIC**

- Cross-sectional and small interventional studies have suggested that physical activity may benefit physical function and symptom burden in multiple sclerosis (MS), but long-term prospective data on disability progression have been lacking.

**WHAT THIS STUDY ADDS**

- Using a population-based incident MS cohort linked to a nationwide clinical registry, we followed over 3200 individuals for up to 15 years and found that physical activity was consistently associated with reduced risk of disability worsening and progression to Expanded Disability Status Scale milestones. Importantly, we provide large-scale evidence suggesting that increasing activity after diagnosis is linked to improved outcomes, while prediagnosis activity was not.

**HOW THIS STUDY MIGHT AFFECT RESEARCH, PRACTICE OR POLICY**

- Our findings support physical activity as a potentially modifiable factor in MS progression. In the context of the limited treatment options for progressive MS, they support the integration of physical activity counselling into standard MS care.

## Background

Multiple sclerosis (MS) is a chronic inflammatory and neurodegenerative disease of the central nervous system characterised by demyelination, axonal injury and gliosis. The aetiology of MS is multifactorial, involving a complex interplay of genetic predisposition and environmental factors.^1^ Among the environmental factors, Epstein-Barr virus infection, low sun exposure, vitamin D deficiency, smoking and obesity have been identified as potential contributors to the development and progression of MS.^1^

Physical activity is widely recognised for its numerous health benefits, including improved cardiovascular health and better overall physical fitness.^2^ In the context of MS, physical activity has been shown to improve mobility and muscle strength,^3 4^ reduce fatigue^5–7^ and enhance health-related quality of life.^6 7^ Despite the known general benefits of physical activity, its impact on risk of disease progression over longer time has been insufficiently explored. Most clinical intervention studies have found that Expanded Disability Status Scale (EDSS) scores remain unchanged over shorter term intervention periods.^8 9^ Cross-sectional studies have generally observed an inverse relationship between physical activity and the level of disability,^8 9^ but the direction of a potential causal relationship is not possible to determine due to the lack of a time dimension.

Understanding how physical activity affects disease progression in MS is important for developing comprehensive management strategies. By identifying the relationship between physical activity levels and disease outcomes, healthcare providers can better prioritise resources and guidance for patients. In the current study, we followed 3284 individuals with MS from an incident case-control study to explore the effects of different levels of physical activity on the progression of MS-related disability.

## Methods

The Epidemiologic Investigation of MS (EIMS) is a Swedish nationwide population-based case-control study aiming at understanding the factors influencing MS aetiology and progression. Between April 2005 and December 2019, the study recruited 3567 newly diagnosed MS patients from hospital-based neurology units and privately run clinics across Sweden, achieving a response rate of 93%. All participants in the study were diagnosed according to the McDonald criteria.^10 11^ On inclusion, participants provided detailed information on environmental exposures and lifestyle habits by filling out a standardised questionnaire. More comprehensive details on the study design and methods are provided elsewhere.^12^ Of the 3567 patients, 3365 (96%) were followed up with EDSS scores in the Swedish MS registry. Patients with incomplete information regarding physical activity were excluded (n=81). The present study thus comprised 3284 patients with MS.

To explore the influence of lifestyle changes post diagnosis, participants were asked to complete a digital follow-up questionnaire in 2021, which captured lifestyle habits from the time of diagnosis. Of the 1823 patients who completed the questionnaire (response rate 67%), 1724 were followed up with EDSS scores in the Swedish MS registry (online supplemental eFigure 1). The study received approval from the Regional Ethical Review Board at Karolinska Institute (reference number 2004-252/1-4 and 2013/1691–32) and was conducted in accordance with the ethical standards set forth in the 1964 Declaration of Helsinki and its later amendments.

10.1136/jnnp-2025-336738.supp1 Supplementary data

## Definition of exposure

Participants were asked to describe their physical activity levels at study inclusion by choosing one of four alternatives for the past year and for 5 years prior. The alternatives were as follows: (1) low physical activity. Predominantly sedentary activities during leisure time, with physical activities such as walking and cycling (usually without sweating) performed for less than 2 hours per week; (2) moderate physical activity. Engaging in physical activities such as walking, cycling, more exertive household work, ordinary gardening activities, fishing, table tennis or bowling (usually without sweating), for at least 2 hours per week; (3) moderate-high physical activity. Regularly exercising for at least 30 min 1–2 times per week, for example, running, swimming, playing tennis or badminton or other activities that induce sweating; (4) high physical activity. Regularly exercising for at least 30 min at least three times per week, for example, running, swimming, playing tennis/badminton, doing aerobics or engaging in other activities that induce sweating. The same question was used in the 2021 follow-up questionnaire, where participants provided data on their physical activity levels in 5-year periods, covering the period between diagnosis and 2021.

## Outcome measures

The Swedish MS registry is used in all neurology units nationwide and is integrated into the clinical documentation system. For each patient, healthcare professionals prospectively record data regarding medical treatment, disease activity and physical functioning.

To analyse disability progression, baseline was defined as the date of the first recorded EDSS. Confirmed disability worsening (CDW) was defined as an increase in the EDSS score, sustained across two visits at least 6 months apart: <1 point or 1.5 points if baseline EDSS was 0 and >0.5 points if baseline EDSS was ≥5.5. Only confirmed events were counted as endpoints, and participants were censored at the time of their first CDW, loss to follow-up or end of study. Analysis of time to reach EDSS milestone scores of 3 and 4, confirmed at two consecutive visits, was limited to patients with a baseline EDSS <3.

### Cerebrospinal fluid neurofilament light ascertainment

In a subset of participants (n=321), cerebrospinal fluid (CSF) neurofilament light (NfL) values within six months of the recorded MS diagnosis date were obtained from the Swedish MS Registry, reflecting measurements performed in routine clinical laboratories using validated immunoassays. Results were recorded in standard concentration units. To reduce skewness, NfL values were log-transformed prior to analysis.

### Directed acyclic graph and covariate selection

We summarised our causal assumptions linking physical activity at diagnosis to long-term disability progression using a directed acyclic graph (DAG) (online supplemental eFigure 2). The minimally sufficient adjustment set implied by the DAG comprised age at diagnosis, sex, ancestry, county, MS phenotype at diagnosis, disease duration at diagnosis, baseline EDSS, body mass index (BMI), smoking, sun exposure and fish consumption. Disease-modifying therapy (DMT) is post baseline and is depicted as a potential mediator rather than a confounder.

## Statistical analysis

Categorical variables were summarised using frequency and percentage. Continuous variables were summarised using mean and SD. To quantify the magnitude of group differences across physical activity levels, we calculated standardised effect sizes. For continuous variables, we used eta squared (η²) from one-way analysis of variance (ANOVA) models. For categorical variables, we used Cramér’s V from χ^2^ tests.

Time-to-event outcomes (24-week CDW, EDSS 3 and EDSS 4) were analysed using multivariable Cox proportional hazard regression to estimate HRs and 95% CIs. Physical activity at diagnosis was modelled as a four-level exposure (low, moderate, moderate-high, high). Participants were right-censored at the last EDSS assessment, death or end of follow-up (06 April 2022), whichever occurred first. The proportional hazard assumption was tested using Schoenfeld residuals, with no violations of proportionality observed. To assess dose-response, physical activity was coded as an ordinal variable (1–4) and entered as a continuous term. We report the HR per one-category increase with its 95% CI. We additionally conducted stratified analysis by clinical (age at diagnosis, sex, disease duration at diagnosis and baseline EDSS) and lifestyle variables (BMI, sun exposure, fish consumption and smoking). For stratification, continuous variables were dichotomised at their medians, whereas sex and smoking used their natural categories.

First, we performed the analyses including all patients to evaluate the overall effect of physical activity on MS progression. Next, we analysed the subgroup of participants who had completed the 2021 follow-up questionnaire (n=1724). We focused on patients who had not changed their level of physical activity between diagnosis and 2021, while still including those who had changed their activity levels in the model. This allowed us to assess the impact of sustained physical activity while accounting for variations in behaviour. Finally, we analysed the subgroup of patients who had changed their activity levels during follow-up. Specifically, we compared patients who increased from low activity with those who maintained a low level, those who increased or decreased from moderate activity with those who maintained a moderate level, and those who increased or decreased from moderate-high activity with those who remained at that level. For patients with initially high activity, we compared those who had decreased their activity with those who remained highly active.

We conducted several sub-analyses to provide a more nuanced understanding of the impact of physical activity on MS progression. First, we performed a sub-analysis focused on patients with relapsing-remitting onset MS, given that this is the most common form of the disease and may respond differently to lifestyle interventions. Additionally, we separately analysed patients of Nordic origin. We conducted a sensitivity analysis excluding individuals with low physical activity, thereby comparing moderate activity against moderate-high and high activity levels. In order to address potential variations in clinical assessments and practices during the recruitment period, we performed a sensitivity analysis that specifically targeted participants enrolled during the peak 5 year period from 2007 to 2011. We separately analysed individuals who reported sensitivity to heat during follow-up. For the NfL subset, we used a general linear model with log-transformed NfL as the outcome to compare levels between low and higher physical activity groups.

Covariate adjustment followed the DAG-derived minimally sufficient set described above. Accordingly, all multivariable models adjusted for age at diagnosis, sex, county (n=21), ancestry (Nordic or non-Nordic), disease phenotype, disease duration, baseline EDSS, BMI, smoking, sun exposure habits and fish consumption habits. We additionally included DMT to obtain treatment-adjusted estimates. DMT exposure was summarised as the proportion of the duration of follow-up spent on DMT. BMI was categorised as underweight, normal weight, overweight or obese using WHO cut-offs. Sun exposure was assessed with three 4-point items on ultraviolet radiation exposure. Scores were summed to an index 3–12. Smoking was coded as current smokers versus non-smokers. Sun exposure was dichotomised into low (value below median) or high (above median). Participants were asked how often, on average, they consumed lean and fatty fish. Responses were recorded on a 4-point scale: never/seldom, 1–3 times/months, weekly or daily. We constructed an index by summing the responses, yielding a value between 2 (lowest exposure) and 8 (highest exposure). Fish consumption was dichotomised into low (value below median) or high (above median). Relapses were not included as covariates in the models, as the aim was to assess the overall association between physical activity and long-term disability progression.

Several variables were considered a priori but were not part of the DAG-derived minimally sufficient adjustment set: having another autoimmune disease than MS (yes or no), passive smoking (ever or never), a history of mononucleosis (yes, no or unknown) and alcohol consumption (categorised into no consumption, low, moderate or high consumption according to cut-offs used by Statistics Sweden.^13^ As a sensitivity check, we added each variable to the primary model. None changed the association between physical activity and MS outcomes, and they were not retained in the final adjustment set. All analyses were conducted in SAS V.9.4.

## Results

We followed 3284 patients with MS for up to 15 years. The mean age at diagnosis and inclusion in EIMS was 38 years (SD 11). Physical activity at baseline was inversely correlated with age, baseline EDSS, disease duration, BMI and smoking, and positively correlated with sun exposure and fish consumption. While many baseline characteristics differed significantly across physical activity groups, most standardised effect sizes were small, indicating limited practical differences. Baseline characteristics of cases of the overall sample and by physical activity levels are presented in table 1.

**Table 1:** Baseline characteristics of overall sample and by physical activity level

	Total	Low	Moderate	Moderate-high	High	P value	Effect size*
N	3284	628	1396	748	512		
Age at diagnosis (SD)	37.7 (11.1)	39.4 (11.5)	38.5 (11.0)	36.8 (10.9)	34.9 (10.4)	<0.0001	0.018
Female, n (%)	2326 (71)	424 (68)	996 (71)	541 (72)	365 (71)	0.2	0.037
Nordic origin, n (%)	2603 (80)	471 (76)	1122 (81)	610 (83)	400 (79)	0.005	0.063
Treatment (SD)	0.9 (0.2)	0.9 (0.2)	0.9 (0.2)	0.9 (0.2)	0.9 (0.2)	0.8	0.070
MS phenotype							
Relapsing, n (%)	3080 (94)	557 (89)	1328 (95)	701 (94)	494 (96)	<0.0001	0.081
Progressive, n (%)	158 (4.8)	59 (9.4)	54 (3.9)	34 (4.6)	11 (2.2)		
Unknown, n (%)	46 (1.4)	12 (1.9)	14 (1.0)	13 (1.7)	7 (1.4)		
Disease duration, years (SD)	2.6 (3.8)	2.9 (3.9)	2.7 (3.9)	2.5 (3.6)	2.0 (3.2)	<0.0001	0.006
Baseline EDSS (SD)	1.8 (1.4)	2.3 (1.7)	1.8 (1.4)	1.6 (1.3)	1.4 (1.2)	<0.0001	0.036
Sun exposure index (SD)	6.2 (1.8)	5.7 (1.8)	6.0 (1.8)	6.5 (1.8)	6.8 (1.8)	<0.0001	0.041
Past IM, n (%)	590 (18)	96 (15)	262 (19)	148 (20)	84 (16)	0.7	0.032
Body mass index (SD)	25.1 (5.1)	26.5 (6.3)	25.4 (5.0)	24.4 (4.4)	23.7 (4.0)	<0.0001	0.009
Current smoking, n (%)	726 (22)	184 (29)	325 (23)	134 (18)	83 (16)	<0.0001	0.108
Fish consumption (SD)	3.9 (1.1)	3.6 (1.1)	3.9 (1.0)	4.0 (1.1)	4.0 (1.1)	<0.0001	0.046
Alcohol (g/week, SD)	43 (67)	44 (73)	42 (61)	43 (72)	44 (70)	0.1	0.001

*Effect sizes are reported as η² (eta squared) for continuous variables and Cramér’s V for categorical variables.

EDSS, Expanded Disability Status Scale; IM, infectious mononucleosis; MS, multiple sclerosis.

During follow-up, 1513 (46%) of the 3284 patients experienced CDW, 760 (29%) of 2554 reached EDSS 3 and 341 (13%) of 2554 reached EDSS 4. The mean follow-up time was 5.9 years (SD 4.4) for CDW, 7.1 years (SD 4.7) for EDSS 3 and 8.4 years (SD 4.6) for EDSS 4. The proportion of patients reaching each milestone decreased with increasing levels of physical activity. Compared with low physical activity, the risk of CDW was 0.82 (95% CI 0.71 to 0.94) for moderate activity, 0.72 (95% CI 0.0.61 to 0.86) for moderate-high activity and 0.64 (95% CI 0.53 to 0.79) for high activity (table 2). Similarly, physical activity was associated with a lower risk of reaching EDSS 3 and EDSS 4. Significant trends indicated a lower risk of EDSS-related outcomes with increasing physical activity (table 2).

**Table 2:** HR with 95% CI of having unfavourable outcomes post diagnosis, by physical activity level at diagnosis

First clinical disease worsening						
	N	Years (SD)	Outcome (%)	HR (95% CI)*	HR (95% CI)†	HR (95% CI)‡
Low	628	5.5 (4.2)	331 (53)	1.0 (reference)	1.0 (reference)	0.86 (0.82 to 0.91)
Moderate	1396	6.0 (4.5)	666 (48)	0.80 (0.69 to 0.91)	0.82 (0.71 to 0.94)	
Moderate-high	748	6.2 (4.2)	323 (43)	0.70 (0.60 to 0.83)	0.72 (0.61 to 0.86)	
High	512	6.3 (4.1)	193 (38)	0.60 (0.50 to 0.72)	0.64 (0.53 to 0.79)	
EDSS 3						
	N	Years (SD)	Outcome (%)	HR (95% CI)*	HR (95% CI)†	
Low	409	6.7 (4.6)	156 (38)	1.0 (reference)	1.0 (reference)	0.85 (0.78 to 0.93)
Moderate	1091	6.9 (4.7)	348 (32)	0.81 (0.67 to 0.99)	0.86 (0.71 to 1.04)	
Moderate-high	617	7.5 (4.7)	158 (26)	0.59 (0.46 to 0.74)	0.68 (0.54 to 0.85)	
High	437	7.4 (4.4)	98 (22)	0.51 (0.39 to 0.67)	0.65 (0.49 to 0.86)	
EDSS 4						
	N	Years (SD)	Outcome (%)	HR (95% CI)*	HR (95% CI)†	
Low	409	8.1 (4.5)	82 (20)	1.0 (reference)	1.0 (reference)	0.81 (0.71 to 0.92)
Moderate	1091	8.4 (4.6)	158 (14)	0.72 (0.54 to 0.94)	0.76 (0.58 to 0.99)	
Moderate-high	617	8.6 (4.7)	67 (11)	0.51 (0.36 to 0.72)	0.60 (0.43 to 0.83)	
High	437	8.3 (4.5)	34 (7.8)	0.38 (0.25 to 0.59)	0.51 (0.34 to 0.76)	

*Crude.

†Adjusted for age at diagnosis, sex, residential area, ancestry, disease phenotype, disease duration, baseline EDSS, disease-modifying therapy, BMI, sun exposure, fish consumption and smoking.

‡Ordinal trend HR; HR per one-category increase in physical activity coded 1–4 (low, moderate, moderate-high, high), estimated from the adjusted Cox model.

BMI, body mass index; EDSS, Expanded Disability Status Scale.

Our findings remained consistent when dichotomised by clinical variables (table 3) and lifestyle variables (table 4). Limiting the analysis to those with normal neurological status (EDSS 0) yielded similar results, with a risk of EDSS progression being 0.67 (95% CI 0.46 to 0.97) among those with high physical activity, compared with those with low activity. Similarly, among those with a baseline EDSS of at least 3, the risk of CDW was 0.65 (95% CI 0.44 to 0.91) among those with a moderate-high activity level and 0.63 (95% CI 0.39 to 1.02) among those with a high activity level, compared with low physical activity.

**Table 3:** HR with 95% CI of CDW post diagnosis, by physical activity level at diagnosis, stratified by clinical variables

First clinical disease worsening (CDW)				
	Female		Male	
	HR (95% CI)*	HR (95% CI)†	HR (95% CI)*	HR (95% CI)†
Low	1.0 (reference)	1.0 (reference)	1.0 (reference)	1.0 (reference)
Moderate	0.87 (0.74 to 1.02)	0.88 (0.74 to 1.02)	0.73 (0.57 to 0.92)	0.71 (0.56 to 0.91)
Moderate-high	0.74 (0.62 to 0.89)	0.74 (0.62 to 0.90)	0.61 (0.46 to 0.80)	0.60 (0.45 to 0.80)
High	0.63 (0.51 to 0.78)	0.66 (0.53 to 0.82)	0.57 (0.41 to 0.79)	0.57 (0.40 to 0.80)
	Age at diagnosis < median (37 years)		Age at diagnosis > median (37 years)	
	HR (95% CI)*	HR (95% CI)†‡	HR (95% CI)*	HR (95% CI)†‡
Low	1.0 (reference)	1.0 (reference)	1.0 (reference)	1.0 (reference)
Moderate	0.88 (0.72 to 1.09)	0.87 (0.70 to 1.08)	0.79 (0.67 to 0.94)	0.84 (0.71 to 1.00)
Moderate-high	0.69 (0.55 to 0.88)	0.66 (0.51 to 0.84)	0.75 (0.61 to 0.91)	0.79 (0.64 to 0.98)
High	0.72 (0.55 to 0.93)	0.69 (0.53 to 0.90)	0.56 (0.43 to 0.73)	0.64 (0.48 to 0.83)
	Disease duration < median (1 year)		Disease duration > median (1 year)	
	HR (95% CI)*	HR (95% CI)†‡	HR (95% CI)*	HR (95% CI)†‡
Low	1.0 (reference)	1.0 (reference)	1.0 (reference)	1.0 (reference)
Moderate	0.83 (0.63 to 1.07)	0.86 (0.67 to 0.95)	0.83 (0.71 to 0.96)	0.83 (0.71 to 0.97)
Moderate-high	0.71 (0.53 to 0.97)	0.76 (0.53 to 0.80)	0.69 (0.58 to 0.83)	0.70 (0.58 to 0.84)
High	0.59 (0.42 to 0.83)	0.64 (0.45 to 0.91)	0.64 (0.52 to 0.78)	0.67 (0.54 to 0.84)
	Baseline EDSS < median (1.5)		Baseline EDSS > median (1.5)	
	HR (95% CI)*	HR (95% CI)†‡	HR (95% CI)*	HR (95% CI)†‡
Low	1.0 (reference)	1.0 (reference)	1.0 (reference)	1.0 (reference)
Moderate	0.93 (0.75 to 1.16)	0.92 (0.73 to 1.15)	0.72 (0.61 to 0.85)	0.82 (0.69 to 0.98)
Moderate-high	0.73 (0.58 to 0.93)	0.72 (0.56 to 0.92)	0.62 (0.51 to 0.76)	0.75 (0.60 to 0.92)
High	0.71 (0.54 to 0.92)	0.71 (0.54 to 0.93)	0.46 (0.35 to 0.59)	0.59 (0.44 to 0.78)

*Crude.

†Adjusted for age at diagnosis, residential area, ancestry, disease phenotype, disease duration, baseline EDSS, disease-modifying therapy, BMI, sun exposure, fish consumption and smoking.

‡Adjusted for sex.

BMI, body mass index; EDSS, Expanded Disability Status Scale.

**Table 4:** HR with 95% CI of CDW post diagnosis, by physical activity level at diagnosis, stratified by lifestyle variables

First clinical disease worsening (CDW)				
	BMI < median (24.2 kg/m^2^)		BMI > median (24.2 kg/m^2^)	
	HR (95% CI)*	HR (95% CI)†‡	HR (95% CI)*	HR (95% CI)†‡
Low	1.0 (reference)	1.0 (reference)	1.0 (reference)	1.0 (reference)
Moderate	0.87 (0.71 to 1.07)	0.82 (0.67 to 1.01)	0.78 (0.64 to 0.94)	0.80 (0.65 to 0.97)
Moderate-high	0.70 (0.56 to 0.88)	0.67 (0.53 to 0.85)	0.76 (0.60 to 0.96)	0.78 (0.62 to 0.99)
High	0.58 (0.45 to 0.75)	0.57 (0.44 to 0.74)	0.61 (0.45 to 0.82)	0.65 (0.47 to 0.88)
	Sun exposure<median		Sun exposure>median	
	HR (95% CI)*	HR (95% CI)†§	HR (95% CI)	HR (95% CI)†§
Low	1.0 (reference)	1.0 (reference)	1.0 (reference)	1.0 (reference)
Moderate	0.82 (0.70 to 0.97)	0.84 (0.72 to 0.99)	0.79 (0.66 to 0.94)	0.83 (0.69 to 1.00)
Moderate-high	0.66 (0.54 to 0.81)	0.70 (0.56 to 0.86)	0.71 (0.58 to 0.89)	0.76 (0.61 to 0.95)
High	0.63 (0.49 to 0.80)	0.68 (0.53 to 0.88)	0.69 (0.54 to 0.90)	0.70 (0.49 to 0.99)
	Fish consumption<median (4)		Fish consumption>median (4)	
	HR (95% CI)*	HR (95% CI)†¶	HR (95% CI)	HR (95% CI)†¶
Low	1.0 (reference)	1.0 (reference)	1.0 (reference)	1.0 (reference)
Moderate	0.89 (0.73 to 1.08)	0.88 (0.72 to 1.07)	0.89 (0.73 to 1.08)	0.88 (0.72 to 1.07)
Moderate-high	0.69 (0.54 to 0.87)	0.71 (0.55 to 0.91)	0.69 (0.54 to 0.87)	0.71 (0.55 to 0.91)
High	0.62 (0.47 to 0.81)	0.64 (0.48 to 0.85)	0.62 (0.47 to 0.81)	0.64 (0.48 to 0.85)
	Current smokers		Non-smokers	
	HR (95% CI)*	HR (95% CI)†**	HR (95% CI)	HR (95% CI)†**
Low	1.0 (reference)	1.0 (reference)	1.0 (reference)	1.0 (reference)
Moderate	0.99 (0.77 to 1.28)	0.92 (0.71 to 1.20)	0.77 (0.66 to 0.90)	0.77 (0.66 to 0.90)
Moderate-high	0.75 (0.54 to 1.03)	0.70 (0.50 to 0.98)	0.67 (0.57 to 0.80)	0.68 (0.56 to 0.80)
High	–	–	0.53 (0.43 to 0.65)	0.55 (0.45 to 0.68)

*Crude.

†Adjusted for age at diagnosis, sex, residential area, ancestry, disease phenotype, disease duration, baseline EDSS and disease-modifying therapy.

‡Adjusted for sun exposure, fish consumption and smoking.

§Adjusted for BMI, fish consumption and smoking.

¶Adjusted for BMI, sun exposure and smoking.

**Adjusted for BMI, sun exposure and fish consumption.

BMI, body mass index; EDSS, Expanded Disability Status Scale.

We also observed similar results when we limited the analyses to participants of Nordic origin (online supplemental eTable 1) and those with relapsing-remitting onset MS (online supplemental eTable 2). The inverse association between physical activity and lower risk of unfavourable EDSS-related outcomes persisted when excluding those with low physical activity, thereby comparing moderate activity with moderate-high and high activity (online supplemental eTable 3).

When physical activity before baseline was compared with 5 years prior to inclusion in EIMS, 880 patients had decreased their physical activity while 388 reported increased activity. There were no signs of association between physical activity level 5 years prior to inclusion in EIMS and EDSS-related outcomes. However, when we excluded individuals who had changed their activity level during the 5 years prior to inclusion, we observed similar results as in our main analysis (data not shown).

### Persistent and changed physical activity during follow-up

Among the 1724 patients who completed the follow-up questionnaire in 2021, 512 patients (27%) had changed their physical activity during the first 2 years of follow-up, with 329 decreasing and 183 increasing their activity. Increasing the activity level from a moderate level was associated with a HR of CDW of 0.59 (95% CI 0.40 to 0.88) compared with maintaining a moderate level. Similarly, increasing the activity level from moderate-high to high was associated with a lower risk of CDW (HR 0.69, 95% CI 0.43 to 0.95) compared with maintaining a moderate-high level (table 5). There was no significant difference in baseline EDSS between those who increased their physical activity level and those who remained at the same level, in either of the activity groups. The relationship between physical activity and favourable outcomes became more pronounced when the analysis was limited to patients who maintained consistent physical activity during the follow-up period (online supplemental eTable 4). The majority of patients developed sensitivity to head during follow-up (53%), however, a sensitivity analysis restricted to those who reported heat sensitivity revealed similar findings (online supplemental eTable 5). The association between physical activity and favourable EDSS-related outcomes also remained consistent when we restricted the analysis to include those recruited between 2007 and 2011 (data not shown). In a subset of individuals with available CSF NfL data collected near diagnosis, individuals with low physical activity had higher CSF NfL levels compared with those with higher activity (p=0.026). Older age was also independently associated with higher NfL (p=0.039), while disease duration and baseline EDSS showed non-significant trends in the expected direction. When the analysis was restricted to individuals with baseline EDSS <2, the association between physical activity and CSF NfL remained statistically significant and became more pronounced (p=0.033) (data not shown).

**Table 5:** HR with 95% CI of having clinical disease worsening post diagnosis among patients with low/moderate physical activity at diagnosis, by physical activity level 2 years post diagnosis

Baseline physical activity=1					
Physical activity 2 years post-diagnosis	N	Years (SD)	Outcome (%)	HR (95% CI)*	HR (95% CI)†
Unchanged	281	6.0 (4.6)	164 (58)	1.0 (reference)	1.0 (reference)
Increased	34	7.0 (3.8)	14 (41)	0.56 (0.33 to 0.97)	0.63 (0.36 to 1.09)
Baseline physical activity=2					
Physical activity 2 years post diagnosis	N	Years (SD)	Outcome (%)	HR (95% CI)*	HR (95% CI)†
Decreased	189	5.7 (4.4)	110 (58)	1.29 (1.03 to 1.62)	1.46 (1.16 to 1.84)
Unchanged	461	6.7 (4.7)	238 (52)	1.0 (reference)	1.0 (reference)
Increased	87	8.2 (4.9)	28 (32)	0.53 (0.36 to 0.78)	0.59 (0.40 to 0.88)
Baseline physical activity=3					
Physical activity 2 years post diagnosis	N	Years (SD)	Outcome (%)	HR (95% CI)*	HR (95% CI)†
Decreased	94	5.6 (3.8)	63 (67)	1.29 (1.03 to 1.62)	1.69 (1.29 to 2.21)
Unchanged	258	7.6 (4.5)	129 (50)	1.0 (reference)	1.0 (reference)
Increased	62	7.6 (4.4)	20 (32)	0.53 (0.36 to 0.78)	0.69 (0.43 to 0.96)
Baseline physical activity=4					
Physical activity 2 years post diagnosis	N	Years (SD)	Outcome (%)	HR (95% CI)*	HR (95% CI)†
Decreased	46	6.2 (4.1)	23 (21)	1.44 (0.91 to 2.28)	1.40 (0.85 to 2.31)
Unchanged	212	7.5 (4.3)	23 (15)	1.0 (reference)	1.0 (reference)

* Crude.

†Adjusted for age at diagnosis, sex, residential area, ancestry, disease phenotype, disease duration, baseline EDSS, disease-modifying therapy, BMI, sun exposure, fish consumption and smoking.

BMI, body mass index; EDSS, Expanded Disability Status Scale.

## Discussion

The extent of engaging in regular physical exercise after diagnosis of MS was inversely related to risk of future disability worsening, with consistent findings across subgroups and in sensitivity analyses. Even moderate physical activity was associated with favourable EDSS-related outcomes, but with a further lowering of risk as physical activity levels increased. Furthermore, while increasing physical activity after being diagnosed with MS was associated with improved outcomes, those who reduced or stopped exercising displayed greater risk of disability worsening, in turn indicating absence of beneficial association between past physical activity level and future disease trajectory. This emphasises the importance of maintaining regular physical activity to continue claiming clinical benefits. Our findings provide valuable knowledge for prioritisation of healthcare resources and support the notion that physical activity should be considered an integral part of comprehensive MS care in clinical practice. Our observation that individuals with higher physical activity at diagnosis had significantly lower NfL concentrations in CSF provides objective support to the observed association between physical activity and more favourable MS outcomes. Although limited to a subset of participants and based on a single time point, this finding suggests that physical activity may be associated with reduced neuroaxonal damage early in the disease course. The association was stronger among individuals with minimal disability at baseline, further reducing the likelihood of reverse causation and supporting the hypothesis that physical activity may modulate neuroaxonal damage before substantial disability has developed.

Our findings align with preclinical MS models, which indicate that physical activity may offer neuroprotective benefits and induce immunomodulatory effects.^14–17^ In contrast, clinical exercise intervention studies generally find unchanged EDSS scores, but are hampered by short study duration and small sample sizes.^8 9^ Also, a smaller longitudinal study with 95 participants with MS followed over 1 year found that those with baseline daily step counts below the cohort median had higher odds of disability worsening during the follow-up.^18^ However, the median EDSS was 4 and almost 40% had progressive MS, which means that it is difficult to dissociate an effect on neuromuscular functioning from that on MS disease progression, at least in the medium term. Furthermore, while cross-sectional surveys also have shown an association between physical activity and MS disability level,^8 9^ any speculation about a causal relationship is made difficult by the study design.

Several factors could contribute to physical activity potentially leading to a more favourable disability trajectory in MS, some of which are not unique to MS, but reflect general neuroprotective mechanisms. As highlighted in a recent review,^19^ exercise supports healthy brain ageing through multiple pathways, including improved cardiovascular health, enhanced cerebral perfusion^20 21^ and increased cortical thickness, particularly in regions critical for cognitive and motor function such as the hippocampus.^22^ Physical activity exerts strong anti-inflammatory and immunomodulatory effects, reduces oxidative stress and improves blood-brain barrier integrity.^16 23^ Additionally, it inhibits the hyperactivation of microglia and astrocytes, limiting microgliosis and astrogliosis and thereby reducing neuroinflammation.^20^ These effects are complemented by increased expression of neurotrophic factors such as brain-derived neurotrophic factors, supporting synaptic plasticity and neuronal survival.^24 25^ Moreover, endurance training influences cellular pathways linked to autophagy, DNA repair and epigenetic modifications that may enhance long-term brain health.^19^

Beyond its biological effects, physical activity supports mental health and helps regulate stress responses, both of which are important for well-being. Maintaining muscle strength, balance and flexibility through exercise may also directly impact mobility and neuromuscular function, mitigating some of the functional decline captured by EDSS scores.

Our study’s strengths include its population-based design with incident cases of MS, a high response rate and detailed exposure information that allows us to account for several potential confounding factors. Furthermore, the long follow-up period and the use of a well-established national registry strengthen the reliability and generalisability of our results.

The information regarding physical activity collected at study inclusion is likely to be subjected to minimal recall bias. However, the classification of physical activity into four broad categories may not capture the full range of activity levels and intensities. A further limitation is that self-reported physical activity typically shows only modest agreement with objective measures, which is characteristic of widely used instruments in large cohorts. Such tools are appropriate for ranking participants into broad activity categories at the population level rather than estimating precise individual levels.^26–29^ Any resulting misclassification is likely non-differential with respect to outcomes and would therefore tend to bias the observed associations towards the null, particularly when comparing extreme groups. Changes in physical activity after baseline were assessed through a follow-up questionnaire sent out in 2021. Since recall error and systematic overestimation are more likely when activity is reported retrospectively, our analyses of changes in physical activity are interpreted cautiously, with the expectation that any remaining recall error would attenuate rather than inflate associations.

Although the response rate was lower in the EIMS follow-up study, the association between physical activity and EDSS-related outcomes remained nearly identical to that of the overall sample, indicating a low degree of selection bias in this step. Moreover, the participants who contributed follow-up data did not differ significantly from those who only participated at baseline with respect to age, sex or baseline EDSS, further reducing concern about attrition bias influencing our results.

We adjusted for a wide range of clinical and lifestyle variables, including age, sex, MS phenotype, disease duration, baseline EDSS, treatment, BMI, sun exposure, fish consumption and smoking status, but not for relapses, as our aim was to capture the impact of physical activity on MS disease activity as a whole. Our findings remained robust across various subgroups, demonstrating consistency in the association between physical activity and favourable EDSS-related outcomes. However, residual confounding by unmeasured variables cannot be ruled out.

Reverse causation is a relevant concern, especially for patients with greater disability at inclusion, which may be the cause of reduced physical activity, thereby potentially leading to an overestimation of the inverse association between activity level and MS progression. We adjusted for baseline EDSS in all models and observed consistent associations in strata defined by baseline disability, including among participants with a baseline normal neurologic status. Associations also remained similar when we restricted the analysis to patients who had maintained their physical activity during follow-up. Among those who completed the follow-up questionnaire, baseline EDSS was similar between those who increased and those who maintained their activity level, indicating that changes in activity were not driven by disease severity at diagnosis. Therefore, we consider it unlikely that reverse causation had a major impact on our results. Finally, although EDSS is primarily a clinician-rated instrument, it includes components that rely partly on patient-reported symptoms. There is some potential for shared variance with self-reported physical activity. However, all EDSS outcomes were defined as sustained changes confirmed across two visits, which reduces the risk that transient or subjective symptoms influenced outcome. Moreover, the potential influence of rating behaviour in epidemiologic studies, where participants report both exposures and outcomes, has been investigated in the context of musculoskeletal disorders. The authors found no evidence of systematic ‘high’ or ‘low’ rating behaviour among individuals when assessing neutral and non-affective variables such as time, weight, physical exposures, pain or other symptoms.^30^ The consistency of our findings for both CDW and objective EDSS milestones suggests that the observed associations are unlikely to be fully explained by shared subjectivity between exposure and outcome. Supporting this, our exploratory analysis of Nfl concentrations showed lower levels among physically active individuals, consistent with reduced underlying disease activity.

In conclusion, our study demonstrates a highly consistent and dose-dependent correlation between physical activity level and risk of future disability worsening, which may be due both to the general positive effects of exercise on brain and body functions, but potentially also specific effects on mechanisms underlying MS disability progression. These findings strengthen the evidence base for providing healthcare resources and encouraging patients with MS to engage in and maintain a high level of physical activity to optimise long term outcomes.

## Data Availability

Data are available upon reasonable request. Anonymised data underlying this article will be shared on reasonable request from any qualified investigator who wants to analyse questions that are related to the published article.
