# Supplementary material for: Beneficial impact of physical activity on multiple sclerosis disability progression
Source: J Neurol Neurosurg Psychiatry. 2025 Sep 23;97(3):209–16. doi: 10.1136/jnnp-2025-336738 (PMC13151437; doi:10.1136/jnnp-2025-336738)
Supplement: Supplementary data [file jnnp-97-3-s001.pdf]

eTable 1. HR with 95% CI of having unfavorable outcomes post-diagnosis, by physical activity level at diagnosis. Nordic origin.

First clinical disease worsening (CDW)						
	N	Years (SD)	Outcome (%)	HR (95% CI)[1]	HR (95% CI)[2]	
Low	471	5.5 (4.2)	251 (53)	1.0 (reference)	1.0 (reference)	0.84 (0.79-0.90)
Moderate	1122	6.0 (4.5)	537 (48)	0.82 (0.71-0.96)	0.83 (0.71-0.96)	
Moderate-high	610	6.3 (4.3)	263 (43)	0.69 (0.58-0.83)	0.70 (0.59-0.84)	
High	400	6.5 (4.1)	143 (36)	0.56 (0.45-0.69)	0.59 (0.48-0.73)	
EDSS 3						
	N	Years (SD)	Outcome (%)	HR (95% CI)[1]	HR (95% CI)[2]	
Low	291	6.8 (4.6)	113 (39)	1.0 (reference)	1.0 (reference)	0.85 (0.77-0.94)
Moderate	872	7.0 (4.6)	278 (32)	0.83 (0.66-1.04)	0.91 (0.72-1.15)	
Moderate-high	130	7.6 (4.8)	130 (26)	0.61 (0.47-0.80)	0.73 (0.56-0.96)	
High	71	7.5 (4.3)	71 (21)	0.50 (0.36-0.68)	0.65 (0.47-0.90)	
EDSS 4						
	N	Years (SD)	Outcome (%)	HR (95% CI)[1]	HR (95% CI)[2]	
Low	291	8.1 (4.4)	64 (22)	1.0 (reference)	1.0 (reference)	0.78 (0.67-0.91)
Moderate	872	8.4 (4.6)	127 (15)	0.67 (0.49-0.92)	0.78 (0.57-1.08)	
Moderate-high	130	8.7 (4.8)	53 (11)	0.45 (0.31-0.67)	0.59 (0.39-0.88)	
High	71	8.3 (4.5)	25 (7.4)	0.35 (0.21-0.56)	0.50 (0.30-0.83)	

[1]crude; [2]adjusted for age at diagnosis, sex, residential area, ancestry, disease phenotype, disease duration, baseline EDSS, disease-modifying therapy, BMI, sun exposure, fish consumption, and smoking.

eTable 2. HR with 95% CI of having unfavorable outcomes post-diagnosis, by physical activity level at diagnosis. Relapsing-remitting onset MS.

First clinical disease worsening (CDW)						
	N	Years (SD)	Outcome (%)	HR (95% CI)[1]	HR (95% CI)[2]	
Low	557	5.6 (4.2)	288 (52)	1.0 (reference)	1.0 (reference)	0.86 (0.81-0.91)
Moderate	1328	6.1 (4.5)	622 (47)	0.84 (0.73-0.96)	0.82 (0.71-0.95)	
Moderate-high	701	6.4 (4.2)	301 (43)	0.71 (0.61-0.84)	0.70 (0.59-0.83)	
High	494	6.3 (4.1)	185 (37)	0.64 (0.53-0.77)	0.65 (0.53-0.78)	
EDSS 3						
	N	Years (SD)	Outcome (%)	HR (95% CI)[1]	HR (95% CI)[2]	
Low	388	6.7 (4.6)	145 (37)	1.0 (reference)	1.0 (reference)	0.86 (0.78-0.93)
Moderate	1056	7.0 (4.7)	330 (31)	0.82 (0.67-1.01)	0.90 (0.72-1.10)	
Moderate-high	594	7.6 (4.7)	149 (25)	0.60 (0.47-0.76)	0.69 (0.54-0.89)	
High	426	7.4 (4.4)	98 (23)	0.55 (0.42-0.72)	0.67 (0.50-0.89)	
EDSS 4						
	N	Years (SD)	Outcome (%)	HR (95% CI)[1]	HR (95% CI)[2]	
Low	388	8.1 (4.4)	73 (19)	1.0 (reference)	1.0 (reference)	0.82 (0.71-0.94)
Moderate	1056	8.5 (4.6)	145 (14)	0.73 (0.54-0.98)	0.81(0.59-1.10)	
Moderate-high	594	8.6 (4.7)	63 (11)	0.54 (0.38-0.78)	0.65 (0.45-0.94)	
High	426	8.3 (4.6)	34 (8.0)	0.43 (0.28-0.67)	0.56 (0.35-0.88)	

[1]crude; [2]adjusted for age at diagnosis, sex, residential area, ancestry, disease phenotype, disease duration, baseline EDSS, disease-modifying therapy, BMI, sun exposure, fish consumption, and smoking.

eTable 3. HR with 95% CI of having unfavorable outcomes post-diagnosis, by physical activity level at diagnosis.

First clinical disease worsening (CDW)						
	N	Years (SD)	Outcome (%)	HR (95% CI)[1]	HR (95% CI)[2]	
Moderate	1396	6.0 (4.5)	666 (48)	1.0 (reference)	1.0 (reference)	0.87 (0.81-0.94)
Moderate-high	748	6.2 (4.2)	323 (43)	0.85 (0.74-0.97)	0.84 (0.74-0.96)	
High	512	6.3 (4.1)	193 (38)	0.74 (0.63-0.87)	0.77 (0.66-0.91)	
EDSS 3						
	N	Years (SD)	Outcome (%)	HR (95% CI)[1]	HR (95% CI)[2]	
Moderate	1091	6.9 (4.7)	348 (32)	1.0 (reference)	1.0 (reference)	0.86 (0.77-0.96)
Moderate-high	617	7.5 (4.7)	158 (26)	0.73 (0.60-0.88)	0.79 (0.65-0.95)	
High	437	7.4 (4.4)	98 (22)	0.65 (0.52-0.82)	0.77 (0.62-0.97)	
EDSS 4						
	N	Years (SD)	Outcome (%)	HR (95% CI)[1]	HR (95% CI)[2]	
Moderate	1091	8.4 (4.6)	158 (14)	1.0 (reference)	1.0 (reference)	0.81 (0.68-0.97)
Moderate-high	617	8.6 (4.7)	67 (11)	0.72 (0.54-0.96)	0.80 (0.59-1.06)	
High	437	8.3 (4.5)	34 (7.8)	0.55 (0.38-0.79)	0.67 (0.46-0.98)	

[1]crude; [2]adjusted for age at diagnosis, sex, residential area, ancestry, disease phenotype, disease duration, baseline EDSS, disease-modifying therapy, BMI, sun exposure, fish consumption, and smoking.

eTable 4. HR with 95% CI of having unfavorable outcomes post-diagnosis, by physical activity level at diagnosis. Unchanged level of training during follow-up.

First clinical disease worsening (CDW)						
	N	Years (SD)	Outcome (%)	HR (95% CI)[1]	HR (95% CI)[2]	
Low	114	5.1 (4.1)	77 (68)	1.0 (reference)	1.0 (reference)	0.84 (0.76-0.92)
Moderate	341	6.6 (4.6)	169 (50)	0.77 (0.62-0.97)	0.79 (0.63-0.99)	
Moderate-high	143	7.2 (3.8)	72 (50)	0.64 (0.49-0.83)	0.68 (0.52-0.87)	
High	130	8.0 (4.5)	45 (35)	0.54 (0.40-0.72)	0.58 (0.43-0.78)	
EDSS 3						
	N	Years (SD)	Outcome (%)	HR (95% CI)[1]	HR (95% CI)[2]	
Low	66	6.6 (5.0)	36 (55)	1.0 (reference)	1.0 (reference)	0.80 (0.71-0.91)
Moderate	273	7.7 (4.7)	97 (36)	0.84 (0.62-1.16)	0.87 (0.67-1.27)	
Moderate-high	120	9.2 (4.5)	26 (22)	0.62 (0.44-0.90)	0.66 (0.48-0.98)	
High	116	9.1 (4.4)	22 (19)	0.51 (0.34-0.77)	0.54 (0.36-0.81)	
EDSS 4						
	N	Years (SD)	Outcome (%)	HR (95% CI)[1]	HR (95% CI)[2]	
Low	66	8.7 (4.6)	22 (33)	1.0 (reference)	1.0 (reference)	0.75 (0.62-0.91)
Moderate	273	9.7 (4.2)	39 (14)	0.54 (0.34-0.85)	0.58 (0.37-0.92)	
Moderate-high	120	10.0 (4.6)	9 (7.5)	0.50 (0.30-0.83)	0.54 (0.33-0.92)	
High	116	10.1 (4.1)	7 (6.0)	0.36 (0.19-0.67)	0.38 (0.21-0.71)	

[1]crude; [2]adjusted for age at diagnosis, sex, residential area, ancestry, disease phenotype, disease duration, baseline EDSS, disease-modifying therapy, BMI, sun exposure, fish consumption, and smoking.

eTable 5. HR with 95% CI of having unfavorable outcomes post-diagnosis, by physical activity level at diagnosis. Respondents with sensitivity to heat.

First clinical disease worsening (CDW)						
	N	Years (SD)	Outcome (%)	HR (95% CI)[1]	HR (95% CI)[2]	
Low	184	5.3 (4.0)	118 (64)	1.0 (reference)	1.0 (reference)	0.87 (0.78-0.96)
Moderate	409	6.2 (4.6)	221 (54)	0.75 (0.60-0.94)	0.77 (0.61-0.97)	
Moderate-high	209	6.5 (4.2)	114 (55)	0.70 (0.54-0.90)	0.71 (0.54-0.94)	
High	117	6.8 (4.3)	59 (50)	0.64 (0.46-0.87)	0.64 (0.46-0.90)	
EDSS 3						
	N	Years (SD)	Outcome (%)	HR (95% CI)[1]	HR (95% CI)[2]	
Low	116	6.8 (4.6)	58 (50)	1.0 (reference)	1.0 (reference)	0.84 (0.74-0.96)
Moderate	306	7.2 (4.7)	132 (43)	0.83 (0.61-1.12)	0.89 (0.66-1.20)	
Moderate-high	170	8.2 (4.6)	63 (37)	0.60 (0.42-0.87)	0.69 (0.48-1.03)	
High	98	8.2 (4.2)	32 (33)	0.54 (0.35-0.83)	0.63 (0.40-0.98)	
EDSS 4						
	N	Years (SD)	Outcome (%)	HR (95% CI)[1]	HR (95% CI)[2]	
Low	116	8.4 (4.2)	36 (31)	1.0 (reference)	1.0 (reference)	0.77 (0.63-0.93)
Moderate	306	9.2 (4.5)	66 (22)	0.63 (0.42-0.95)	0.63 (0.40-0.96)	
Moderate-high	170	9.6 (4.4)	30 (18)	0.48 (0.30-0.79)	0.49 (0.30-0.82)	
High	98	9.3 (4.2)	16 (16)	0.47 (0.26-0.85)	0.48 (0.26-0.89)	

[1]crude; [2]adjusted for age at diagnosis, sex, residential area, ancestry, disease phenotype, disease duration, baseline EDSS, disease-modifying therapy, BMI, sun exposure, fish consumption, and smoking.

eFigure 1. Study flow and analysis population

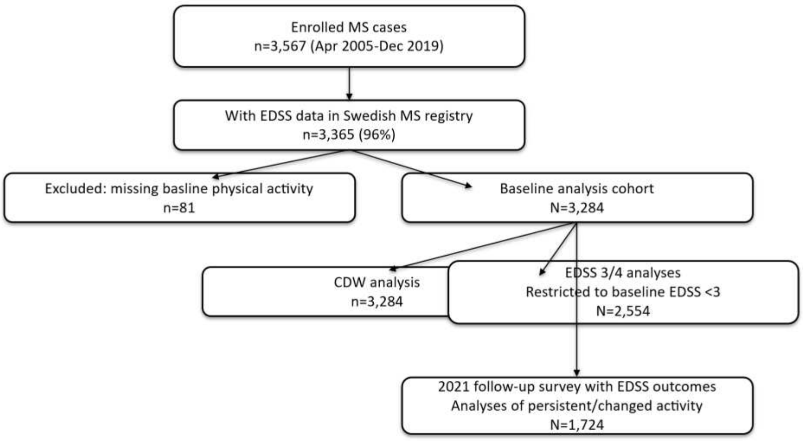

CDW=confirmed disability worsening; EDSS=Expanded Disability Status Scale.

eFigure 2. Directed acyclic graph (DAG) for the association between physical activity at diagnosis and long-term disability progression in MS.

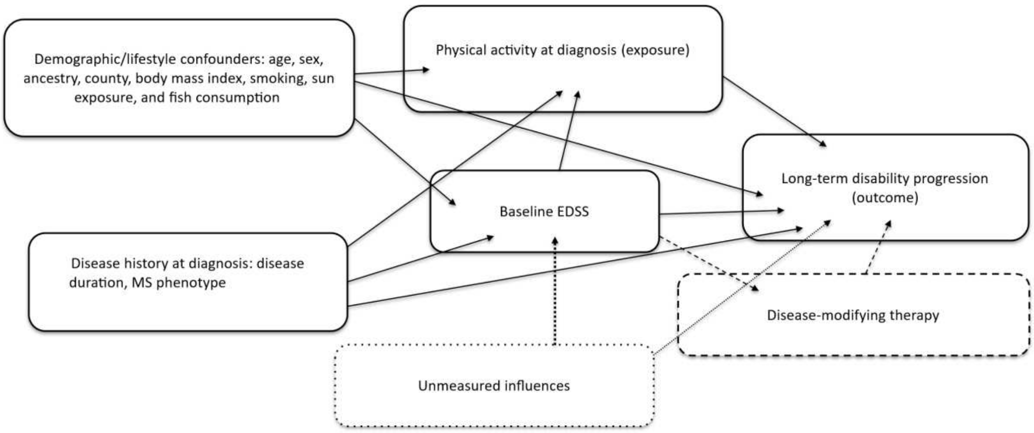

Solid line=directed edge; dashed=post-baseline treatment (mediator); dotted=unmeasured (latent) influences.

The minimally sufficient adjustment set includes age at diagnosis, sex, ancestry, county, disease duration at diagnosis, MS phenotype at diagnosis, baseline EDSS, BMI, smoking, sun exposure, and fish consumption. DMT exposure is shown as post-baseline treatment and may partially lie on the causal pathway.
